# Surgical Technique for Removal of Old Universal Slotted AO Femoral Nail: A Case Report

**DOI:** 10.1155/2024/5603392

**Published:** 2024-10-03

**Authors:** Naoko Onizuka, Brenton Douglass, Marc Swiontkowski

**Affiliations:** ^1^ Department of Orthopedic Surgery University of Minnesota, Minneapolis, Minnesota, USA; ^2^ TRIA Orthopedics, Bloomington, Minnesota, USA; ^3^ Park Nicollet Methodist Hospital, St. Louis Park, Minnesota, USA

## Abstract

This paper presents a surgical technique for the removal of an old universal femoral nail preceding total hip arthroplasty (THA) in a 50-year-old male patient with left hip osteoarthritis. The patient had undergone femur nail insertion approximately 35 years ago. Due to the necessity of nail removal prior to THA, surgery to remove the nail was planned. There are challenges posed by the design of the old universal femoral nail system, particularly its side slot which made engagement of the conical bolt difficult. The successful removal of the nail was eventually achieved, enabling subsequent THA. Individuals who received this old implant years ago may now require its removal as part of osteoarthritis treatment. Given the lack of familiarity among surgeons with this outdated implant, this paper is aimed at providing essential guidance and insights regarding its removal procedure. This literature represents the inaugural documentation of the surgical technique for the removal of an aged femur nail.

## 1. Introduction

The annual number of hip arthroplasties is increasing combined with the aging population worldwide [1, 2]. In patients who have undergone femoral nail insertion, the removal of the femoral nail is frequently deemed necessary before advancing to total hip arthroplasty (THA), particularly as these patients age and experience hip osteoarthritis. The AO (Arbeitsgemeinschaft für Osteosynthesefragen)/ASIF (the Association of the Study of Internal Fixation) universal nail system was introduced in 1986 and has long been discontinued [3]. This particular nail boasts a thin wall thickness, engineered for flexibility [3]. Its fully slotted open-section cloverleaf design adds complexities to its removal process. This paper explores a unique case, shedding light on the surgical technique used to remove an outdated universal femoral nail system. Given the lack of usage of the AO/ASIF universal nail system in modern orthopedic procedures, this case presents an exceptional opportunity to document the removal procedure.

## 2. Case Presentation

**Figure 1:**
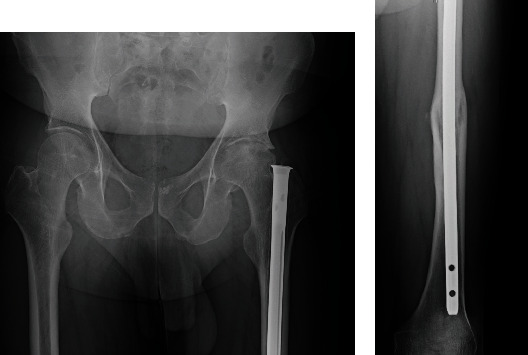
Anteroposterior radiograph showing a universal femoral nail and left hip osteoarthritis (a, b).

A 50-year-old male with a left femur fracture treated with a femoral nail insertion 35 years ago presented with symptomatic left hip osteoarthritis (Figure 1). Given the patient's desire for THA, the nail's removal was deemed necessary prior to the THA surgery.

### 2.1. Surgical Technique

**Figure 2:**
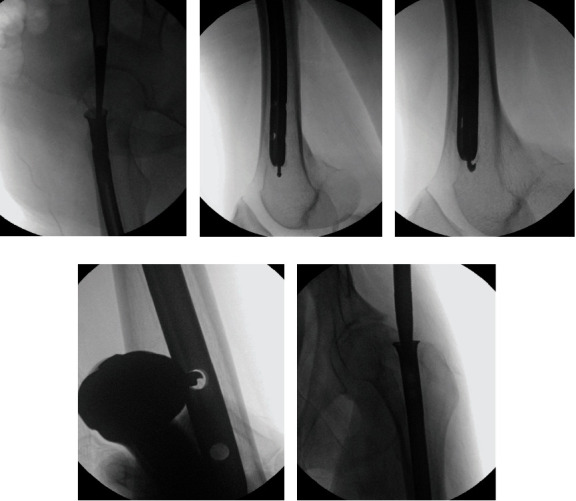
Intraoperative fluoroscopy images. (a) Large curette at the nail entry site. (b) A ball tip guide wire was passed through the nail. (c) Hook wire was passed through the nail. (d) Perfect circle technique to drill the interlock holes. (e) Conical bolt was inserted into the nail.

Under general anesthesia, the patient was positioned laterally using a half bean bag on a Jackson table. General anesthesia was chosen in case muscle paralysis was required during the surgery and to accommodate the potentially prolonged duration of the procedure. A 5-cm incision was made over the greater trochanter, followed by subcutaneous tissue dissection to expose the nail. Bone overgrowth within the nail was removed using a large curette (Figure 2(a)). A ball-tip guide wire was placed through the nail's center (Figure 2(b)), followed by attempts at removal using a conical bolt and backslap hammer, which initially failed, potentially due to insufficient removal of osseous overgrowth and bone growth in the interlock screw holes. Additional strategies involved inserting a hook wire and employing multiple guide wires to enhance engagement of the hook wire (Figure 2(c)). Despite these efforts, nail removal remained unsuccessful, necessitating further measures. Additional incisions were made anteriorly on the distal thigh to access interlock holes. Drill was used at the interlock holes (Figure 2(d)). Then, dissection around the nail's entry point and complete removal of osseous overgrowth were performed using a 1/2-inch osteotome. Subsequent attempts with a conical bolt and backslap hammer finally led to successful nail removal, albeit with noted challenges posed by the nail's design (Figure 2(e)).

## 3. Discussion

Several techniques have been described for removing intramedullary nails, including broken nails or dissociated femoral intramedullary magnetic lengthening nails [4–21]. Some reported details of extractor compatibility with modern femur nails, or minimally invasive technique of femoral nail extraction [22–26]. However, this report represents the sole available documentation concerning this old universal femoral nail system featured by a fully slotted open-section cloverleaf design within our knowledge.

**Figure 3:**
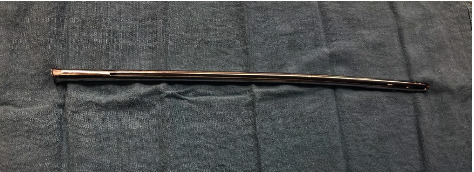
The universal femoral nail, featured by a fully slotted open-section cloverleaf design.

The presented case underscores the importance of meticulous preoperative planning and adaptability during surgery when faced with the challenge of removing old femoral nails. In this instance, the patient had undergone femoral nail insertion approximately 35 years prior, necessitating the development of a tailored surgical technique to address the unique challenges posed by the antiquated nail design. The universal femoral nail, featured by a fully slotted open-section cloverleaf design, which presented difficulties in engaging the conical bolt for removal (Figure 3).

In the setting of old femur nail removal, several challenging situations can arise, such as the conical bolt failing to engage with the nail, the nail being completely incarcerated, or the occurrence of an iatrogenic fracture or injury. Failure to attach the extractor can happen secondary to bone overgrowth, stripping of the proximal threads or, less commonly, due to impacted broken metal device pieces. To overcome these challenges, Georgiadis, Heck, and Ebraheim described a technique using a high-speed drill with a carbide metal cutting bit to create a slot in the proximal end of the nail, enabling the attachment of a hook for subsequent removal [27]. More recently, Pathrot, Mannual, and Shettar reported the effectiveness of the stainless-steel wire technique [28].

When the extractor is unable to be attached, a combination of techniques such as using a hook wire [7], distally impacted multiple guide wires [15], a Hohmann retractor inserted through an access channel in the lateral femoral cortex (push-out technique) [29], ender nail [30], or custom-made devices should be utilized. Those techniques can be effective but cannot be used in solid nails. Some previously reported techniques include using a distal approach where retrograde mobilization of the nail is achieved by tapping it from an infrapatellar position [25] or longitudinal osteotomy [31, 32]. Goosen and Van Hellemondt managed a similar case by cutting the proximal end of the nail to the required length to implant a hip arthroplasty on top of the nail [33]. Overall, a variety of innovative and adaptable strategies are necessary to address the complexities involved in the removal of old femur nails.

In addition, special consideration should be given to patients who received intramedullary nail insertion in low- and middle-income countries (LMICs). The Surgical Implant Generation Network (SIGN) provides intramedullary nails free of charge to hospitals in LMICs for the treatment of long bone fractures, making these procedures more accessible [34]. However, Okwesili et al. reported a high rate of nail extractor device mismatch in these settings, highlighting additional challenges such as limited availability of compatible removal tools, variability in nail designs, and a lack of standardized surgical protocols [35]. These factors can complicate the removal process and require tailored solutions to address the unique circumstances found in LMICs.

In our case, persistence and adaptability eventually led to successful nail removal, enabling subsequent THA. This case highlights the importance of intraoperative decision-making and the ability to modify surgical techniques as needed to overcome challenges. The primary objective of this case report is to disseminate the surgical methodology employed in addressing this challenging scenario.

## 4. Conclusion

This case study highlights a tailored surgical approach for removing an antiquated femoral nail prior to THA in a patient with hip OA. Despite encountering challenges posed by the nail's design, successful removal was ultimately achieved. Such cases emphasize the necessity of a customized surgical strategy to address the unique challenges posed by antiquated orthopedic implants.
